# sRNA scr5239 Involved in Feedback Loop Regulation of *Streptomyces coelicolor* Central Metabolism

**DOI:** 10.3389/fmicb.2019.03121

**Published:** 2020-01-23

**Authors:** Franziska Engel, Elena Ossipova, Per-Johan Jakobsson, Michael-Paul Vockenhuber, Beatrix Suess

**Affiliations:** ^1^Synthetic Genetic Circuits, Department of Biology, Darmstadt University Technology, Darmstadt, Germany; ^2^Division of Rheumatology, Department of Medicine, Karolinska Institutet, Karolinska University Hospital, Solna, Sweden

**Keywords:** sRNA, *Streptomyces*, DasR, carbon metabolism, phosphoenolpyruvate, PEPCK

## Abstract

In contrast to transcriptional regulation, post-transcriptional regulation and the role of small non-coding RNAs (sRNAs) in streptomycetes are not well studied. Here, we focus on the highly conserved sRNA scr5239 in *Streptomyces coelicolor*. A proteomics approach revealed that the sRNA regulates several metabolic enzymes, among them phosphoenolpyruvate carboxykinase (PEPCK), a key enzyme of the central carbon metabolism. The sRNA scr5239 represses *pepck* at the post-transcriptional level and thus modulates the intracellular level of phosphoenolpyruvate (PEP). The expression of scr5239 in turn is dependent on the global transcriptional regulator DasR, thus creating a feedback loop regulation of the central carbon metabolism. By post-transcriptional regulation of PEPCK and in all likelihood other targets, scr5239 adds an additional layer to the DasR regulatory network and provides a tool to control the metabolism dependent on the available carbon source.

## Introduction

Streptomycetes are Gram-positive, filamentous, GC-rich, soil-dwelling bacteria. They are best known for their complex life cycle and their ability to produce a wide range of secondary metabolites. These include two thirds of all known antibiotics but also other bioactive compounds such as immunosuppressants, antivirals, and herbicides (Flärdh and Buttner, 2009). This biochemical potential is associated with a large genome size (usually 7–12 Mbp) and requires an equally large repertoire of regulatory proteins to control the network and adapt to changing environmental conditions (Bentley et al., 2002). *Streptomyces coelicolor* serves as a model organism for this large group of bacteria. Its regulatory repertoire includes ∼12% of its protein coding genes but also a large number of regulatory RNAs are known. The function of most of them, however, remains unknown (Pánek et al., 2008; Swiercz et al., 2008; D’Alia et al., 2010; Vockenhuber et al., 2011; Moody et al., 2013; Jeong et al., 2016; Setinova et al., 2017).

Small non-coding RNAs (sRNAs), approximately 50–500 nucleotides (nts) in length, are found in a broad range of bacteria and play an important role in the post-transcriptional regulation. Most small non-coding RNA act by base-pairing with their target mRNAs, which may affect both stability and/or translation of the target mRNA in a positive or negative manner (reviewed in Romby and Wagner, 2012). Depending on their genomic context, sRNAs are divided into *trans*-encoded regulatory RNAs, which are encoded at different genomic locations from their target genes and often share only limited complementarity with them, and *cis*-encoded (antisense) regulatory RNAs. They are transcribed opposite to annotated genes and share complete complementarity with their targets. Most knowledge about sRNAs, their targets and regulatory networks has so far been derived from Gram-negative bacteria such as *Escherichia coli* and *Salmonella* (Frohlich et al., 2012; Guo et al., 2014; Porcheron et al., 2014; Kim et al., 2019). In contrast, only a few sRNAs in streptomycetes have been experimentally characterized so that their function is known. Examples include scr4677, which is thought to impact the actinorhodin production under specific growth conditions (Hindra et al., 2014) and scr3097, which in combination with a riboswitch influences *rpf*A expression post-transcriptionally (St-Onge and Elliot, 2017). Furthermore, there are the antisense RNA cnc2198.1 that regulates glutamine synthase *glnA* (D’Alia et al., 2010) and scr5239. The latter was identified using a deep sequencing approach (Vockenhuber et al., 2011). scr5239 expression is constitutive under several stress and growth conditions but dependent on the nitrogen supply. It is conserved in two thirds of all currently available *Streptomyces* genomes. The 159 nt long sRNA consists of five stem-loops P1–P5, of which stem P4 is involved in the interaction with both currently known target mRNAs. These targets – the genes for the methionine synthase *metE* and the agarase *dagA* – are crucial for both primary and secondary metabolisms, as they are important for methionine synthesis and the degradation and utilization of agar as a carbon source. Whereas *S. coelicolor* is the only known *Streptomyces* species that carries the agarase gene *dagA*, *metE* is conserved in a wide number of streptomycetes (Vockenhuber et al., 2011; Vockenhuber and Suess, 2012; Vockenhuber et al., 2015). Since non-coding RNAs are known to often control more than one target, and because of its remarkable conservation, we aimed to identify further targets of scr5239. Our previous studies indicated that in contrast to the majority of the characterized sRNAs to date, scr5239 did not induce degradation of the both validated target mRNAs *metE* and *dagA* (Vockenhuber et al., 2011; Vockenhuber et al., 2015). Therefore, we decided to carry out a proteomics study to identify new targets controlled by scr5239.

Here, we present the characterization of a new sRNA target that resulted from the proteomics study, the phosphoenolpyruvate carboxykinase (PEPCK, SCO4979). PEPCK is a key enzyme of the primary metabolism as it connects glycolysis with the tricarboxylic acid (TCA) cycle and is thought to catalyze the first step of gluconeogenesis in all organisms. In the presence of GTP it catalyzes the conversion of oxaloacetate (OAA) to phosphoenolpyruvate (PEP). This reaction is the rate-limiting step in the metabolic pathway that produces glucose from lactate and other precursors derived from the TCA cycle (Delbaere et al., 2004). Here we show that scr5239 controls PEPCK and thus the level of the metabolite PEP. scr5239 itself is controlled by DasR, one of the most important pleiotropic regulators of the primary and secondary metabolism in *S. coelicolor*. It controls more than 50 genes including the biosynthetic pathways for several antibiotics (Świątek-Połatyńska et al., 2015), developmental control (Rigali et al., 2006) or the response to cold shock stress (Nazari et al., 2013). We propose that scr5239 adds an additional layer to the DasR regulatory network by creating a feedback loop regulation for the control of the central metabolism.

## Materials and Methods

### Cultivation of *S. coelicolor*

For growth on solid medium, 10^8^ spores per 300 μl were pregerminated (Kieser et al., 2014), plated out on R2YE with cellophane overlays and incubated at 30°C for 3–4 days. For growth in liquid medium, 10^8^ spores per 50 ml were pregerminated and incubated at 28°C under continuous shaking for 3–4 days in Jasenka medium (10% sucrose, 3% tryptic soy broth, 0,1% MgCl_2_ and 0,1% yeast extract).

### Plasmids and *S. coelicolor* Strains

A list of all plasmids used may be found in Table 1. All integrating plasmids were constructed based on pAR933a (Rodríguez-García et al., 2005). It contains origin of replication for maintenance in *E. coli*, an apramycin resistance gene for selection in *E. coli* and *S. coelicolor*, an integrase gene and attachment site of the phiC31 phage and an origin of transfer. The plasmids used here all enable a stable integration of one single copy per chromosome (Kuhstoss et al., 1991), avoiding possible problems associated with the copy number of multicopy plasmids. The luciferase gene was excised from pAR933a using *Xba*I and *Spe*I and a 3x FLAG-tag (for western blot detection) was inserted, thus converting the *Xba*I site to an *Mfe*I site. Then, an insert containing two *Bsa*I sites for golden gate cloning was cloned into the *Spe*I/*Mfe*I digested vector resulting in pGold_F3. *E. coli* ET12567/pUZ8002 was used to transfer the plasmids into *S. coelicolor* via intergeneric conjugation (Kieser et al., 2014). A list of *S. coelicolor* strains used may be found in Table 2.

**TABLE 1:** List of plasmids used in this study.

**Plasmid**	**Characteristics**	**References**
pAR933a	integrating plasmid	Rodríguez-García et al., 2005
pFT241	pFT74-derivative for DasR overexpression	Schlicht, 2003
pGold_F3	integrating plasmid, with FLAG-Tag to detect integrated genes	This work
pPEPC_F3	plasmid for integrating and detecting PEPCK (5416135–5418114)	This work
pSP4_PEPC_F3	plasmid for integrating and detecting PEPCK, natural promoter replaced by the synthetic Promoter SP4	This work
pPEPC_F3 M1	plasmid for integrating and detecting PEPCK, part of the original 5′ UTR replaced by the metH 5′ UTR	This work
pPEPC_F3_scr5239+	plasmid for integrating and detecting PEPC, with scr5239 in control of the SF14 promoter	This work
pPEPC_F3_scr5239+_M1	plasmid for integrating and detecting PEPC, with a mutated scr5239 in control of the SF14 promoter	This work
pGUS_pscr5239	plasmid for integrating scr5239 promoter and detecting its activity using *gusA*	This work
pGUS_pscr5239_M1	plasmid for integrating a shortened scr5239 promoter variant and detecting its activity using *gusA*	This work
pGUS_pscr5239_M2	plasmid for integrating a scr5239 promoter with mutated *dre* site and detecting its activity using *gusA*	This work

**TABLE 2:** List of *S. coelicolor* strains used in this study.

**Strain**	**Features**	**References**
M145	SCP1^–^, SCP2^–^	Kieser et al., 2014
Δscr5239	M145scr5329::KanR	Vockenhuber and Suess, 2012
scr5239+	M145scr5239p::SF14p	Vockenhuber and Suess, 2012
M145 [pFT241]	M145 [pFT241]	Schlicht, 2003
BAP29	M145dasR::aacC4	Rigali et al., 2006
FE01	M145::pPEPC_F3	This work
FE02	Δscr5239::pPEPC_F3	This work
FE03	scr5239+::pPEPC_F3	This work
FE04	M145::pSP4_PEPC_F3	This work
FE05	Δscr5239::pSP4_PEPC_F3	This work
FE06	scr5239+::pSP4_PEPC_F3	This work
FE07	M145::pPEPC_F3_M1	This work
FE08	Δscr5239::pPEPC_F3_M1	This work
FE09	scr5239+::pPEPC_F3_M1	This work
FE11	Δscr5239::pPEPC_F3_scr5239+	This work
FE12	Δscr5239::pPEPC_F3_scr5239 +_M1	This work
FE13	M145::pGUS_pscr5239	This work
FE14	M145::pGUS_pscr5239_M1	This work
FE15	M145::pGUS_pscr5239_M2	This work

### Mass Spectrometry-Based Quantitative Proteomics

For quantitative liquid chromatography mass spectrometry (LC-MS/MS) analysis, the *S. coelicolor* M145 and the sRNA overexpression and deletion strains (scr5239+ and Δscr5239, respectively) where grown on solid R2YE medium as described above. Cells where harvested at the end of exponential growth when the mycelium just started to turn red. Cell lysis and whole proteome preparation were done as described in section “SDS-PAGE and Western Blot Analysis.”

Proteins were precipitated from the lysates using ReadyPrep^TM^ 2-D Cleanup Kit (Bio-Rad). Obtained protein pellets were suspended in dissolving buffer containing 2% SDS, 8 M urea, 25 mM HEPES, 1 mM DTT (pH 7.4) and the protein concentration was measured using Bradford assay (Bio-Rad). For the digestion, aliquots containing 200 μg of total protein amount were added onto Microcon-10 kDa Centrifugal Filter Units (Millipore) and processed using FASP protocol (Wisniewski et al., 2009) with modified digestion buffer containing 0.2% sodium deoxycholate, 0.25 M urea, in 100 mM HEPES, pH 7.6. After digestion, sodium deoxycholate was removed from the samples by precipitation in the presence of formic acid followed by centrifugation at 12,000 × *g*. Concentration of obtained peptides was measured using Lowry assay. Equal amount of protein digests (100 μg) from each sample were labeled with Tandem Mass Tag (TMT) 6-plex Isobaric Mass Tagging Kit (Thermo Fisher Scientific) according to the manufacturer’s instructions. Labeled samples were dissolved in loading buffer (3% ACN, 0.1% FA), resulting in a final concentration of 10 μg/μl prior to LC-MS/MS analysis. Before analysis on the Q Exactive (Thermo Fischer Scientific), peptides were separated using an Agilent 1200 nano-LC system. Samples were trapped on a Zorbax 300SB-C18, and separated on a NTCC-360/100-5-153 (Nikkyo Technos, Ltd.) column using a gradient of A (5% DMSO, 0.1% FA) and B (90% ACN, 5% DMSO, 0.1% FA), ranging from 5 to 37% B in 240 min with a flow of 0.4 μl/min. The Q Exactive was operated in a data-dependent manner, selecting top five precursors for fragmentation by HCD. The survey scan was performed at 70,000 resolution from 300 to 1700 m/z, with a max injection time of 100 ms and target of 1 × 10^6^ ions. For generation of HCD fragmentation spectra, a max ion injection time of 500 ms and AGC of 1 × 10^5^ were used before fragmentation at 30% normalized collision energy, 35,000 resolution. Precursors were isolated with a width of 2 m/z and put on the exclusion list for 70 s. Single and unassigned charge states were rejected from precursor selection.

Acquired MS raw files were searched against the UniProtKB/*S. coelicolor* database and filtered to a 1% FDR cut off. Ion mass tolerance of precursors equals ±10 ppm, whereas the fragments have a more limited mass tolerance of 0.02 Da for HCD-FTMS. The search algorithm assessed tryptic peptides with maximum one missed cleavage; carbamidomethylation (C), TMT 10-plex (K, N-term) as fixed modifications and oxidation (M) as variable modification. Only unique peptides in the data set were used for quantification, while reporter ions were quantified by Proteome Discoverer on HCD-FTMS tandem mass spectra (integration tolerance 20 ppm).

### SDS-PAGE and Western Blot Analysis

A total of 1 ml liquid culture or 100 mg mycelium from solid medium were harvested and mixed with 1 ml 1× ZAP (50 mM NaCl, 50 mM Tris–HCl pH 8.0, 10% glycerol, 10 mM PMSF). The subsequent disruption of the cells was done using 200 μl glass beads (0.4 mm diameter) and the FastPrep-24 instrument (MP Biomedicals) for 4 × 1 min at 5.5 m/s. Mycelial debris was removed by centrifugation at 4°C. Protein concentration of the supernatant was determined by Bradford assay (Bradford, 1976). Twenty to fifty μg of crude extract were separated by a 6% or 12% SDS-PAGE. 0,5% 2,2,2-trichloroethanol (TCE) in the polyacrylamide gels allowed fluorescent detection of proteins using the ChemiDoc^TM^ MP Imaging System (Bio-Rad). Gels were blotted using the Trans-Blot^®^ Turbo^TM^ Transfer System (Bio-Rad). Membranes were blocked for 1 h in 2% ECL^TM^ Blocking Agent (GE Healthcare) in TBS-T (20 mM Tris base, 150 mM NaCl and 0.1% Tween 20), followed by incubation with a Monoclonal ANTI-FLAG^®^ M2 antibody (Sigma-Aldrich #F1804; 1:50000 in 2% ECL^TM^ Blocking Agent in TBS-T) for 1 h at RT and 3 × 10 min wash in TBS-T. Blots were developed using ECL^TM^ Select Western Blotting Detection Reagent (GE Healthcare) and signals detected with the ChemiDoc^TM^ MP Imaging System. Protein bands were quantified using Image Lab Software 6.0.1. Total protein was used as loading control.

### RNA Isolation

Total RNA was isolated following Vockenhuber et al. (2015). In brief, 1 ml culture or ∼100 mg mycelium were harvested and resuspended in 300 μl lysis buffer (10 mM sodium acetate, 150 mM sucrose, pH 4.8). Glass beads (200 μl, 0.4 mm diameter) and 300 μl acidic phenol were added. Cells were disrupted using a FastPrep-24 instrument (MP Biomedicals) for 4 × 1 min at 5.5 m/s. After phenol/chloroform extraction and ethanol precipitation, the RNA was resuspended in 500 μl ddH_2_O and the concentration was determined (usually 2–4 μg/μl). One hundred μg of total RNA were incubated with 30 U Turbo DNase (Ambion) for 1 h to remove residual DNA, subsequently precipitated and resuspended in 50 μl ddH_2_O. The yield was usually a concentration of 1–1.5 μg/μl, of which 1 μg was quality-checked on a 1% agarose gel.

### RT-qPCR

A total of 1 μg RNA was reverse-transcribed using the SuperScript IV Reverse Transcriptase (Thermo Fisher Scientific) and random hexamers according to the manufacturer’s protocol. After transcription, the 20 μl reaction was filled up to 200 μl with ddH_2_O. mRNA levels were analyzed using 5 μl of 1:1 diluted cDNA product, the Fast SYBR Green PCR Master Mix (Applied Biosystems) and specific primers for the *pepck* (PEPCK_fwd and PEPCK_rev). The genes for *hrdB* (Primers: hrdB_fwd and hrdB_rev) and SCO1544 (Primers: 1544_fwd and 1544_rev) served as an endogenous control (Li et al., 2015). RT-qPCR was carried out on a StepOnePlus machine. All oligonucleotides used may be found in Table 3.

**TABLE 3:** List of oligonucleotides used in this study.

**Primer**	**Sequence (5′–3′)**
PEPCK_fwd	GTCACCGAGTCCTTCGACTG
PEPCK_rev	GATCTTCGGCAGCTTGGACT
hrdB_fwd	AGTTCGAAGCCTCCACTTGT
hrdB_rev	TGCTGACGCCGCTACTCGT
1544_fwd	TCGAGGTCGCCCGGGAACT
1544_rev	GATCACGTAGGTGGGGGTGCC
scr5239_A	CCGGTCGGCCGAAGGATG
5S_A	CGAAATGTAACCGGGCGTTT

### β-Glucuronidase Measurement

The β-glucuronidase gene (*gusA*) was used to determine promoter activity in *S. coelicolor* (Myronovskyi et al., 2011). Here, 10–40 mg of protein crude extract (cell disruption protocol, see section “SDS-PAGE and Western Blot Analysis”) obtained from mycelium harvested from solid medium were used for the enzymatic reaction. The assay was performed as previously described (Rudolph et al., 2015).

### Measurement of PEP Concentration

The PEP Colorimetric/Fluorometric Assay Kit from Sigma-Aldrich was used to determine the PEP concentration in *S. coelicolor* according to the manufacturer’s protocol. 2–4 mg of mycelium harvested from solid medium were used for the enzymatic reaction. Signals were quantified by fluorescence using a Tecan infinite 200 pro multiwell plate reader.

### Northern Blot Analysis

A total of 30 μg total RNA was separated on 6% denaturing polyacrylamide gels and transferred to a positively charged nylon membrane (Hybond-N+, GE Healthcare) in a tank blotting device (Peqlab) at 4°C. As probe for detection, 10 pmol oligonucleotide were radiolabeled at the 5′ end using 5 μl γ-[^32^P] ATP (∼3.3 pmol/μl, Hartmann-Analytic) and 1 μl T4 polynucleotide kinase (Roche) in the supplied buffer for 1 h at 37°C and subsequently purified using Illustra MicroSpin G-25 columns (GE Healthcare). 25 μl radiolabeled oligonucleotides (approximately 300 kc.p.m./μl) where used as probe for each experiment. Signals were quantified by phosphoimaging using a FLA-5000 phosphoimager (Fujifilm life Science). Expression of scr5239 (probe: scr5239_A) was normalized to the amount of 5S rRNA (probe: 5S_A).

### Bioinformatics

#### RNAhybrid

RNAhybrid (Kruger and Rehmsmeier, 2006) is a tool for the prediction of microRNA/target duplexes. It predicts multiple potential binding sites of microRNAs in large target mRNAs by finding the energetically most favorable hybridization sites of a small RNA in a large RNA. To predict potential binding sites, we used RNAhybrid Version 2.2 with default parameters. Judging from past experience, the predicted mRNA/sRNA interaction sites are more reliable with this program than with other available prediction tools.

#### Target Explorer and PREDetector

For prediction of *dre* sites, an evaluation matrix ranking the individual nucleotides of the binding site according to their importance was required. For this purpose, the program Target Explorer was used (Sosinsky et al., 2003). To create the position matrix, the sequences of five different known *dre* sites were used. These can be found in Supplementary Table S1. The position matrix thus created is shown in Supplementary Figure S1. Using this matrix and PREDetector (Hiard et al., 2007), we predicted 15 new *dre* sites in the genome of *S. coelicolor*. The results, ranked according to the degree of matching with the matrix (score), can be found in Supplementary Table S2. The *dre* site upstream of scr5239 ranked fourth of all predicted sites.

## Results

### Quantitative Proteomics Reveals 32 Proteins That Show scr5239-Dependent Changes in Expression

**FIGURE 1:**
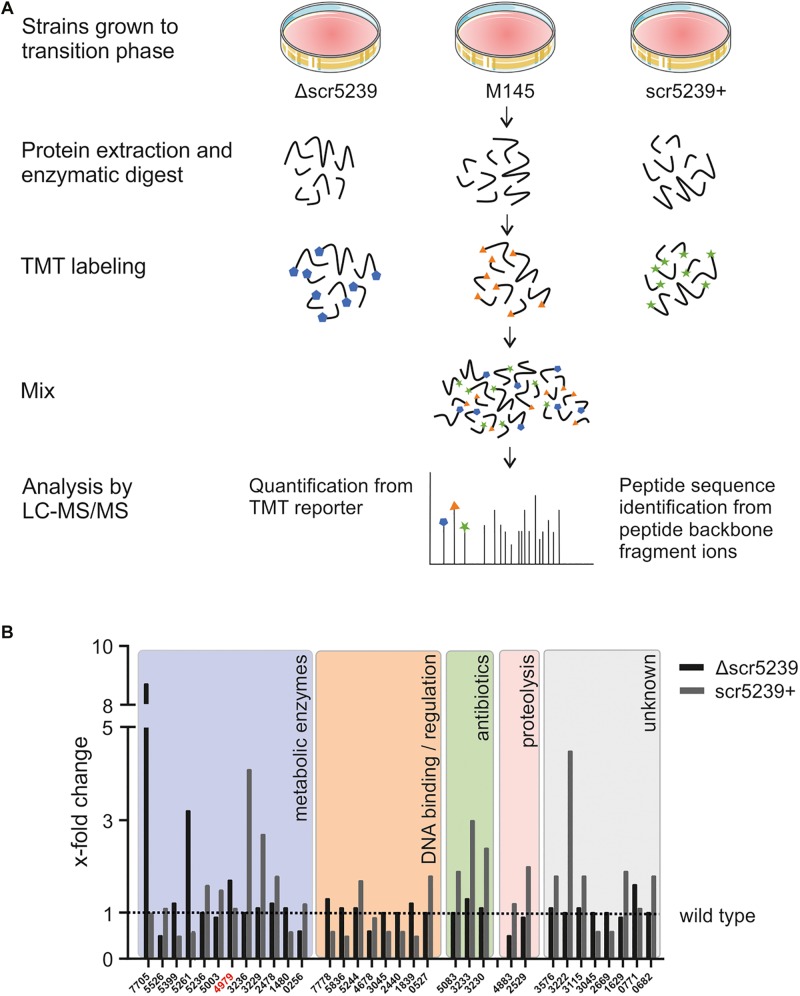
Quantitative proteomics analysis of three different *S. coelicolor* strains. **(A)** LS-MS/MS workflow. *S. coelicolor* Δscr5239, M145, and scr5239+ were grown on rich media and harvested in the transition phase. The samples of the three strains were labeled with different TMT labels. The peptides of the three strains where pooled and the fragments where analyzed in tandem MS. As a result, a fragment of the TMT label is cleaved of each peptide. The mass of the cleaved part of the label differs with each label type. In consequence, we were able to quantify the relative amount of each peptide fragment in the input strains. The signal strength of the cleaved-off part of the labels was then used for input normalization. Thus, this method allows relative quantification of proteins. **(B)** Results of the quantitative proteomics and subsequent functional analysis. Bars indicate the x-fold change in protein expression in the Δscr5239 and scr5239+ strain compared to the wild type. Only hits with a minimum change of 50% in expression are shown (Δ vs. wt or wt vs. +). Colored boxes show categorization of proteins depending on their functional characteristics. Numbers on the *x*-axis refer to the SCO numbers of the expressed genes. The SCO number of PEPCK is highlighted in red.

To identify new targets of scr5239, we compared the proteome of *S. coelicolor* wild type (M145) with its respective scr5239 deletion (Δscr5239) and overexpression (scr5239+) strain. We applied quantitative proteomics based on the stable isotope labeling method to all three strains grown on R2YE solid medium and harvested in the transition phase. After protein extraction and fragmentation, the peptides where labeled with a different TMT labels for each strain and analyzed using LC-MS/MS (Figure 1A). This method has already been successfully applied in *S. coelicolor* to characterize differentiation and activation of the secondary metabolism (Rioseras et al., 2018).

A total of 1669 proteins were found to be expressed in all three strains (Supplementary Table S3). However, only 32 of them showed a significant change in expression level depending on scr5239, i.e., at least a two-fold change in mass spec. signal strength (Δscr5239 vs. M145 or M145 vs. scr5239+) (Figure 1B). Functional analysis revealed that potential targets of the sRNA cluster in five main groups. The largest group, comprising about half of the identified annotated proteins (12), are metabolic enzymes. In addition to that, eight proteins cluster in the group of DNA-binding and regulatory proteins. Another three identified proteins are part of the CDA antibiotic biosynthesis cluster and two are involved in proteolysis. The remaining eight proteins are of unknown function. Figure 1B shows the detected changes in protein expression upon deletion or overexpression of the sRNA along with a functional analysis.

Since metabolic enzymes were the most abundant, we focused on potential targets within this group. Moreover, the fact that the previously identified targets of this sRNA, *metE* and *dagA* (Vockenhuber et al., 2011, 2015) play a role in central carbon and amino acid metabolism further encouraged us to prioritize metabolic enzymes. The candidate with the most interesting functionality here was SCO4979, which encodes the PEPCK. It connects glycolysis with the TCA cycle and catalyzes the rate-limiting step in the gluconeogenese. In consequence, it is of key importance for the central carbon metabolism. We therefore continued with this candidate.

### PEPCK Protein and mRNA Levels Are Dependent on scr5239 Expression

**FIGURE 2:**
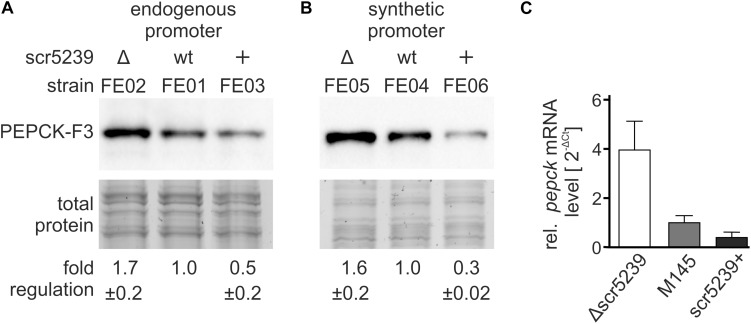
Expression analysis of PEPCK in different strains. The strains were grown on rich media and harvested in the transition phase. **(A)** Western blot analysis using FE01, FE02, and FE03 to detect PEPCK-F3 under control of its endogenous promoter. **(B)** Western blot analysis using FE04, FE05, and FE06 to detect PEPCK-F3 under control of the synthetic promoter SP4. **(C)** RT-qPCR results. mRNA levels of *pepck* were detected in *S. coelicolor* M145, Δscr5239, and scr5239+ and normalized to the levels in M145. Measurements were normalized to the respective strain expressing wild type scr5239; error bars represent the standard deviation calculated from three independent experiments.

To investigate the role of scr5239 in the regulation of PEPCK, we initially analyzed the mRNA and protein levels of PEPCK depending on the scr5239 level. To validate the quantitative proteomics data, we performed western blot analyses. We used an integrating plasmid and fused a 3x FLAG-tag to the C-terminus of PEPCK resulting in pPEPCK_F3. We attached the tag to the C-terminus as sRNA/mRNA interactions usually take place in the 5′UTR or close to the 5′ end of the coding region of the mRNA. The plasmid was integrated into the genome of wild type (M145), Δscr5239 and scr5239+ into the attachment site of the phiC31 phage, thus resulting in strains FE01, FE02, and FE03, respectively. A list of all strains used and constructed across all experiments may be found in Table 2. We used the same growth conditions as in the TMT-based experiment and harvested the cells in the transition phase. The western blot analysis shown in Figure 2A effectively confirms the TMT data with a two-fold increase in PEPCK expression in FE02 (Δscr5239) and approximately two-fold decrease in FE03 (scr5239+) compared to the wild type (FE01). To exclude an effect on the transcription of PEPCK, we replaced the wild type *pepck* promoter in pPEPCK_F3 with the synthetic promoter SP4 (Horbal et al., 2018), thus generating the integrating plasmid pSP4_PEPCK_F3. Integration resulted in the respective strains FE04, FE05, and FE06 for M145, Δscr5239, and scr5239+, respectively. Western blot analyses of these strains show similar regulation, which indicates that the observed regulation is not promoter-dependent and therefore taking place on the posttranscriptional level (Figure 2B). mRNA levels of *pepck* were measured in M145, Δscr5239 and scr5239+. The RT-qPCR results revealed a four-fold increase of *pepck*-levels in Δscr5239 and a slight decrease (0.6-fold) in scr5239+ compared to the levels in M145 (Figure 2C).

Most known sRNAs base pair with the translational initiation region (TIR) and thus initiate mRNA degradation (Saramago et al., 2014). The results of the western blot and RT-qPCR analyses indicate that the PEPCK regulation by scr5239 follows this mechanism.

### Deletion of scr5239 Leads to an Increase of the Energy Donor Phosphoenolpyruvate in *S. coelicolor*

**FIGURE 3:**
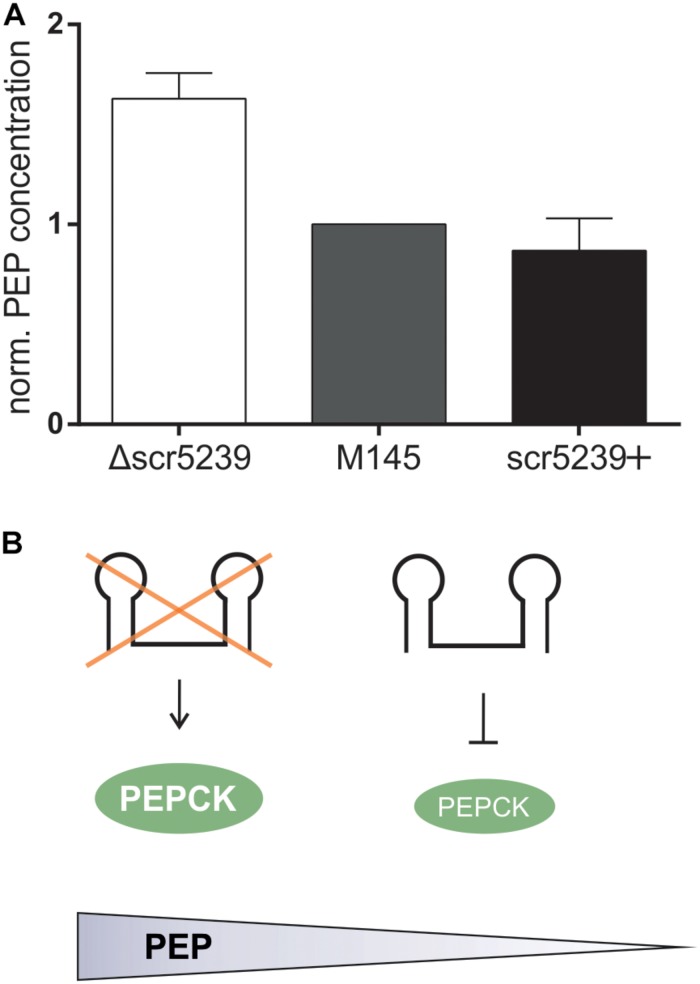
PEP levels in M145, Δscr5239, and scr5239+. **(A)** PEP levels were determined in *S. coelicolor* M145, Δscr5239, and scr5239+ and normalized to the levels in M145; error bars represent the standard deviation calculated from three independent experiments. **(B)** Schematic overview of the connection between scr5239, PEPCK, and PEP levels in *S. coelicolor*.

Next, we aimed to investigate the metabolic impact of PEPCK regulation through scr5239. We determined the steady state level of PEP, the product converted by PEPCK from OAA. M145, Δscr5239, and scr5239+ were grown and harvested under the same conditions as for the quantitative proteomics analysis. As shown in Figure 3, deletion of scr5239 dramatically modulates the cellular PEP level. In Δscr5239 we were able to detect an increase of 70% in PEP level compared to M145. Increasing the scr5239 level (scr5239+) did not significantly alter the PEP level (Figure 3A). Figure 3B illustrates the influence of the sRNA on PEPCK and consequently on the level of PEP.

Phosphoenolpyruvate is a key intermediate in carbohydrate metabolism and involved in glycolysis and gluconeogenesis. Its metabolization to pyruvic acid by pyruvate kinase generates ATP and is vitally important for the energy balance of the cell, as PEP has the highest-energy phosphate bond found in organisms. The influence the sRNA exerts on the cellular concentration of this key metabolite indicates its crucial role in the regulation of the primary metabolism.

### Interaction of PEPCK and scr5239

**FIGURE 4:**
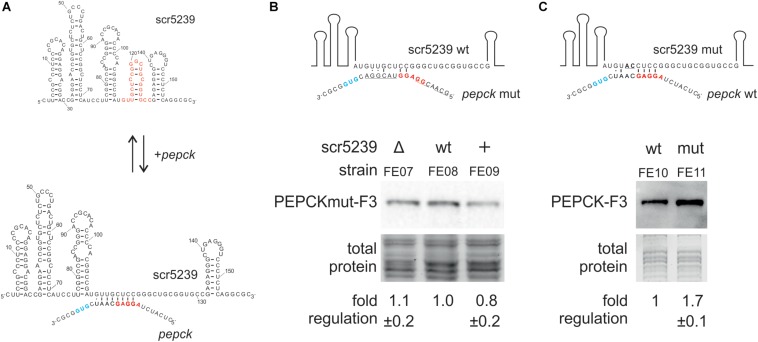
Analysis of the interaction of scr5239 and PEPCK mRNA. **(A)** Predicted interaction site. The sequence of the sRNA is shown completely, that of the *pepck* mRNA only partially. The RBS (red) and the start codon (blue) of *pepck* are highlighted. The interaction site of *dagA* and *metE* is highlighted in red on the sRNA. **(B)** PEPCK was mutated at the predicted interaction site (PEPCmut-F3) and integrated in the genome of the M145, Δscr5239, and scr5239+. Mutated bases in the sequence are underlined. Western blot analysis using FE07, FE08, and FE09 and thus detecting PEPCmut-F3. **(C)** scr5239 was mutated at the predicted interaction site. Mutated bases in the sequence are underlined. The mutated and the wild type scr5239 in combination with PEPC-F3 were integrated into Δscr5239. Western blot analysis using FE10 and FE11 and thus detecting with PEPC-F3. Measurements were normalized to the respective strain expressing wild type scr5239; errors represent the standard deviation calculated from three independent experiments.

To mechanistically characterize the regulation of PEPCK by scr5239, we analyzed the interaction between the sRNA and the target mRNA. We used RNAhybrid (Kruger and Rehmsmeier, 2006) to identify potential interaction sites. RNAhybrid predicted an 8 nt region within stem P4 of the sRNA to interact with the TIR of the *pepck* mRNA (Figure 4A). In consequence, the interaction site with PEPCK predicted for scr5239 was in the same region of the sRNA as the binding sites of the other two targets *dagA* and *metE*.

To verify the predicted interaction site, we introduced mutations within the PEPCK sequence that disrupted potential base pairing. We used plasmid pPEPCK_F3 and exchanged the PEPCK 5′UTR including the ribosomal binding site (RBS) for an 5′UTR of an unrelated gene (methionine synthase MetH), resulting in pPEPCK_F3 M1. The methionine synthase MetH is not regulated by the sRNA (Vockenhuber et al., 2015). Figure 4B illustrates the relevant sequence for *pepck* mRNA. The construct pPEPCK_F3 M1 was integrated into the genome of the three strains M145, Δscr5239, and scr5239+, resulting in FE07, FE08, and FE09. Figure 4B shows that the expression levels of PEPCK are comparable for all three strains. PEPCK expression is not influenced by the scr5239 level as in the corresponding strains FE01, FE02, and FE03 that harbor a functional wild type PEPCK 5′UTR (compare Figure 2B). Hence, the mutated PEPCK-F3 does not show dependency on the sRNA.

Furthermore, we mutated scr5239 and analyzed the influence on PEPCK expression. We introduced two mutations (U113A, G114C) into the predicted interaction site of the sRNA. U113 and G114 are central in the predicted binding motif and should prevent both interaction and, consequently, regulation. Figure 4C illustrates the relevant sequence of the mutated sRNA. We designed two variants of a plasmid carrying the PEPCK-F3 sequence and the sequence for scr5239. The former, pPEPC_F3_scr5239+, encoded for the wild type scr5239, the latter, pPEPC_F3_scr5239+_M1, encoded for scr5239 with the described mutations. These constructs were integrated in Δscr5239 resulting in FE10 and FE11. The strains express the sRNA exclusively from the integrated plasmid (expression level of the scr5239 was confirmed by Northern blot, data not shown). Western blot analyses show that the PEPCK is more abundant in the strain with the mutated sRNA than in the strain with the wild type sRNA (Figure 4C), which indicates that the sRNA represses PEPCK expression more strongly than the mutated sRNA. These results suggest that the mutated sRNA is no longer able to bind *pepck* mRNA.

In sum, the results of the mutational analysis confirmed the predicted interaction site of scr5239 with the PEPCK mRNA.

### Regulation of scr5239 by DasR

DasR is a global transcriptional regulator of the metabolism in *S. coelicolor* with more than 50 validated target genes. It is one of the most important pleiotropic regulators of the primary and secondary metabolism in this organism. It controls the *N*-acetylglucosamine metabolism, the monomer unit of the polymer chitin and the primary carbon and nitrogen source for *S. coelicolor* (Rigali et al., 2006; Colson et al., 2007). DasR binds to so called DasR responsive elements (*dre* sites) and prevents the transcription of downstream genes (Rigali et al., 2006; Colson et al., 2007; Rigali et al., 2008; Świątek-Połatyńska et al., 2015). After binding its ligand glucosamine-6-phosphate or *N*-acetylglucosamine, respectively, DasR releases the DNA and transcription can take place. Using PREDetector software (Hiard et al., 2007), we identified a potential *dre* site upstream of scr5239. As the findings of this study clearly indicate a role of the scr5239 in the central metabolism, we decided to explore whether scr5239 is a direct target of DasR.

**FIGURE 5:**
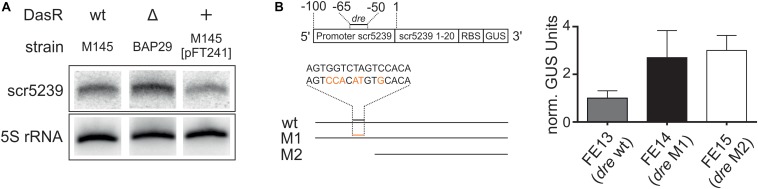
Regulation of scr5239 by DasR. **(A)** Northern blot of scr5239 with RNA harvested from strains with different DasR expression levels. **(B)** Reporter gene measurements of different variants of the scr5239 promoter fused to a *gusA* gene. **(A)** Scheme of the used constructs. The *dre* site of scr5239 is indicated. The sequences of the wild type and the mutated *dre* site are shown. The mutated bases are highlighted in red. **(B)** Results of the reporter gene measurements for FE13, FE14, and FE15. *dre*: DasR responsive element, wt: scr5239 Promoter with wild type *dre* site, M1: scr5239 Promoter with mutated *dre* site, M2: First 50 bp of scr5239 Promoter without *dre* site. Measurements were normalized to FE13; error bars represent the standard deviation calculated from three independent experiments.

First, we analyzed whether scr5239 expression is dependent on DasR. We compared the expression of scr5239 in M145 with a strain lacking the *dasR* gene (BAP29; Rigali et al., 2006 and a strain overexpressing *dasR* (M145 [pFT241]; Schlicht, 2003). Northern blot analysis showed clear DasR-dependent changes in scr5239 expression (Figure 5A). Deletion or overexpression of DasR lead to significant increase and decrease of scr5239 expression, respectively. We then validated the significance of the *dre* site of scr5239 by reporter gene measurements. The scr5239 promoter along with the first 20 bases of the sRNA was fused to a *gusA* reporter gene (pGUS_pscr5239). Two mutants were constructed, one with 6 nt of the *dre* site exchanged (pGUS_pscr5239_M1) and a second one that contained only the first 50 bp of the promoter with the *dre* site deleted (pGUS_pscr5239_M2, Figure 5B, left). The constructs (pGUS_pscr5239, _M1, _M2) were integrated into the genome of M145 resulting in the strains FE13, FE14, and FE15. The strains were grown to the transition phase and the GUS activity was measured. Both mutations lead to an approximately three-fold increase in Δ-glucuronidase activity when compared to the wild type promoter (Figure 5B, right).

The Northern blot analysis and the reporter gene measurements of the promoter activity confirm that DasR regulates scr5239 transcription by interacting with the upstream *dre* site. However, please note that DasR, even when overexpressed, does not fully repress scr5239. Consequently, there are likely more factors that influence the expression rate of the sRNA *in vivo*.

## Discussion

Starting from proteomics data, we confirmed *pepck* as a new target of scr5239 in *S. coelicolor*. Consequently, the sRNA has now three validated targets, which renders it the best characterized sRNA in streptomycetes to our knowledge.

The binding site of scr5239 in the *pepck* gene is located between the RBS and the start codon of the mRNA. In contrast, the binding sites of the other two known scr5239 targets are located within the open reading frame (*dagA* + 33 – + 52, *metE* + 7 – + 30). Furthermore, for *dagA* and *metE*, only the protein levels are changing while their mRNA levels stay constant. However, in the case of PEPCK, mRNA levels change in the same pattern as the protein levels, which is common for mRNA/sRNA interactions. The regulation described here thus follows the conventional mechanism described for the majority of sRNAs, mostly characterized in the well-studied organisms *E. coli* and *Salmonella* (Wagner and Romby, 2015). They regulate the expression of their target genes by blocking access to the RBS and/or start codon, which impedes translation and initiates mRNA degradation.

In the present study, we were able to show that scr5239 is involved in the regulation of the central metabolism of *S. coelicolor*. PEPCK is a key enzyme of the primary metabolism that catalyzes the conversion of OAA to PEP, the first and rate-limiting step of the gluconeogenesis (Delbaere et al., 2004). Since the regulation of glycolysis and gluconeogenesis is mutually exclusive, the control of PEPCK must be exactly adapted to the metabolic demand/situation of the cell. We show that scr5239 is involved in this regulation. It binds to the *pepck* mRNA leading to a reduced level of the enzyme. Consequently, a reduced level of the sRNA significantly increases the intercellular PEP level (see deletion studies). PEP can then be further converted into fructose-1,6-bisphosphate toward glucose but is also involved in carbon uptake and signaling through the phosphotransferase system (PTS) (Bruckner and Titgemeyer, 2002).

Most sRNAs are transcriptionally controlled by σ- or transcription factors. We were able to show that this is also the case for scr5239, which is regulated by the global transcription regulator DasR. We identified a functional *dre* site in the promoter of scr5239. Our findings match the results of previously performed ChIP-on-chip experiments and EMSAs (Świątek-Połatyńska et al., 2015). They showed that the *dre* site of scr5239 was one of the most tightly bound targets of DasR. Moreover, DasR binding to the *dre* site of scr5239 could be observed in the samples taken during vegetative growth and sporulation, which was only the case for a small fraction of the found targets. Furthermore, the genomic locations of DasR and scr5239 provide further insight. DasR is encoded in ORF5231, which puts protein and sRNA in a close evolutionary context. These findings indicate that scr5239 is at least one of the most preferred targets of DasR, thus suggesting a key role of the sRNA in the DasR regulatory network.

**FIGURE 6:**
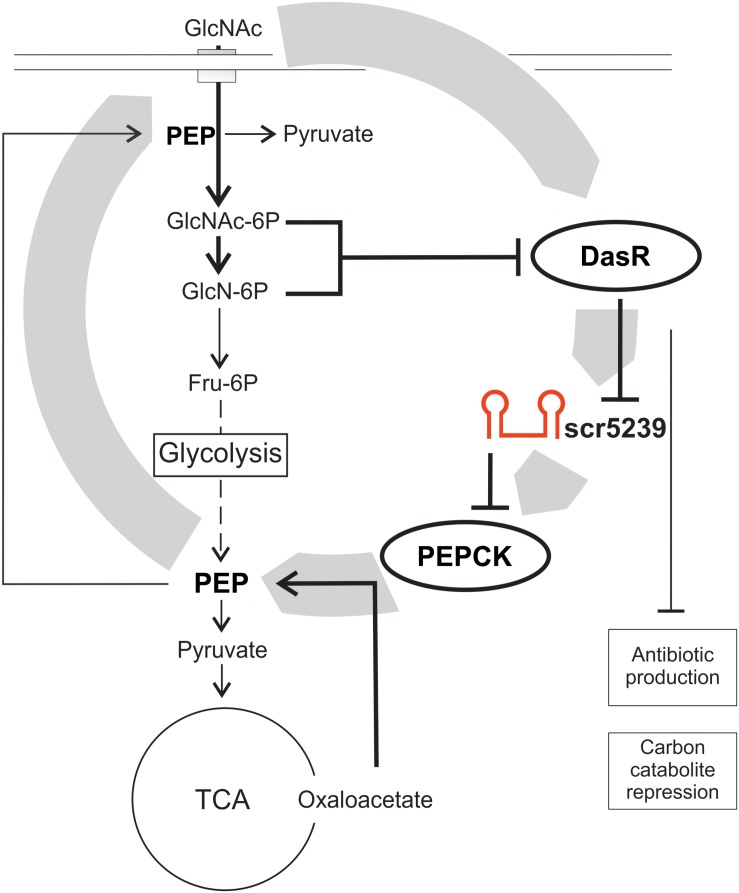
Model of the scr5239 feedback loop. scr5239 is regulated by the global transcriptional regulator DasR that also regulates the antibiotic production and the carbon catabolite repression. scr5239 inhibits the expression of PEPCK and thus influences the PEP levels. Higher PEP levels lead to an enhanced GlcNAc uptake, which as a result inhibits DasR.

Small non-coding RNAs are often involved in forming (negative) feedback loops with the transcription factors they regulate (reviewed in Wagner and Romby, 2015). A feedback loop regulation can also be described for the scr5239 regulation by DasR. DasR is regulated by the PTS in streptomycetes with GlcNAc as preferred carbon source (Nothaft et al., 2003). If the supply of nutrients is sufficient (high level of GlcNAc that is converted via GlcN6-P into Fru-6P as input to glycolysis), DasR in its GlcN6-P-bound conformation cannot act as repressor, hence the sc5239 level is high. As a result, gluconeogenesis is switched off by binding and repressing *pepck* via scr5239, the first and rate-limiting step of gluconeogenesis. If the nutrient supply deteriorates, DasR in its free form can act as repressor and the level of scr5239 decreases. In consequence, the PEP level increases, which initiates gluconeogenesis. However, PEP is not just important as intermediate for glycolysis and gluconeogenesis, but although for carbon uptake via the PTS. Increased PEP levels allow the cells to take up more GlcNAc through the PTS, which leads to a higher concentration of GlcN-6-P. GlcN-6-P in turn represses DasR, which then again leads to higher levels of scr5239. Through this feedback loop scr5239 can to influence its own repressor. A model of such feedback loop regulation including the DasR and scr5239 is depicted in Figure 6. Such regulatory feedback loops have already been described for sRNAs. An example is the transcription factor OmpR that activates the sRNAs OmpA and OmpB whereas these inhibit OmpR (Brosse et al., 2016).

The carbohydrate metabolism is often governed by complex regulatory networks. They allow the survival of bacteria in ever-changing habitats with fluctuating nutrient supplies by rapid adaptation of their metabolic capabilities. Such metabolic networks not only exploit transcription regulation but also include posttranscriptional regulation e.g., the control by sRNAs. Within the last years, sRNAs emerged as an important additional layer to fine-tune regulatory response. sRNAs are proposed to limit the response to substrate availability, set the threshold concentration or module the delay time for activation or shutdown of the system (Durica-Mitic et al., 2018). Prominent examples are the sRNA Spot42 that is involved in the carbon catabolite repression in *E. coli* or the sRNA CsrB and CsrC that control the RNA-binding protein CsrA – a posttranscriptional regulator responsible for the switch between glycolysis and gluconeogenesis. These examples impressively show that bacterial carbohydrate metabolism is controlled at all levels by large and densely interconnected regulatory networks. In *Streptomyces*, DasR is the major hub for the control of the central carbohydrate metabolism. We identified scr5239 as additional player in this regulatory network representing a switch for the cell to decide between gluconeogenese and glycolysis.

## Data Availability Statement

The datasets generated for this study can be found in the PRIDE repository project accession: PXD016811.

## Author Contributions

BS, M-PV, and FE contributed to the conception, design of the study, and wrote the manuscript. FE and M-PV performed most of the experiments. EO and P-JJ were responsible for quantitative proteomics analysis.

## Conflict of Interest

The authors declare that the research was conducted in the absence of any commercial or financial relationships that could be construed as a potential conflict of interest.

## Supplementary Material

FIGURE S1 *dre* site prediction matrix.

TABLE S1 *dre* sites that were used to create the position martix.

TABLE S2 Predicted *dre* sites.

TABLE S3 Quantitative proteomics results.
