# Supplementary material for: sRNA scr5239 Involved in Feedback Loop Regulation of Streptomyces coelicolor Central Metabolism
Source: Front Microbiol. 2020 Jan 23;10:3121. doi: 10.3389/fmicb.2019.03121 (PMC7025569; doi:10.3389/fmicb.2019.03121)
Supplement: FIGURE S1 — dre site prediction matrix. [file Data_Sheet_1.PDF]

Score matrix:

**A**	1,00	-0,22	-1,79	-1,79	-1,79	-1,79	-1,79	-1,79	1,21	-1,79	1,21	-1,79	-1,79	0,73	-1,79	-1,79
**C**	-1,79	-0,14	-1,79	-1,79	-1,79	-1,79	0,45	-1,79	-1,79	-1,79	-1,79	1,30	1,30	-1,79	-0,14	-1,79
**G**	-1,79	0,82	-1,79	1,09	1,30	-1,79	0,82	-1,79	-1,79	1,30	-1,79	-1,79	-1,79	-1,79	1,09	-1,79
**T**	-0,22	-1,79	1,21	-0,22	-1,79	1,21	-1,79	1,21	-1,79	-1,79	-1,79	-1,79	-1,79	0,36	-1,79	1,21
