# Supplementary material for: sRNA scr5239 Involved in Feedback Loop Regulation of Streptomyces coelicolor Central Metabolism
Source: Front Microbiol. 2020 Jan 23;10:3121. doi: 10.3389/fmicb.2019.03121 (PMC7025569; doi:10.3389/fmicb.2019.03121)
Supplement: TABLE S1 — dre sites that were used to create the position martix. [file Table_1.DOCX]

**Supplementary Table 1: *dre* sites that were used to create the position martix**

| **Gen** | ***dre* site** |
| --- | --- |
| nagE1 | actggtgtagaccagt |
| nagE2 | agtggtgtagacctgt |
| ptsH | agttgtctagaccagt |
| dasA | aatggtgtagaccagt |
| crr | tgtggtctagacctct |
