# Supplementary material for: sRNA scr5239 Involved in Feedback Loop Regulation of Streptomyces coelicolor Central Metabolism
Source: Front Microbiol. 2020 Jan 23;10:3121. doi: 10.3389/fmicb.2019.03121 (PMC7025569; doi:10.3389/fmicb.2019.03121)
Supplement: TABLE S2 — Predicted dre sites. [file Table_2.docx]

**Supplementary Table 2: Predicted *dre* sites**

| **Score** | ***dre* site** | **Position** | **Downstream gene** | **Function of the gene** |
| --- | --- | --- | --- | --- |
| 16,97 | actggtctacaccatt | -105 | SCO5232 | sugar transporter |
| 13,99 | acaggtctaaaccatt | -101 | SCO3563 | synthetase |
| 13,97 | actggtctacaccctt | -171 | SCO7263 | chiF, chitinase |
| 12,19 | agtggtctagtccaca | -65 | scr5239 | sRNA scr5239 |
| 11,55 | agtggcgtacacctgt | -214 | SCO7250c | unknown |
| 11,18 | agtggactatacctgt | -245 | SCO6004c | ATP/GTP binding protein |
| 10,90 | agaggtctagacaaaa | -117 | SCO6300c | secreted hydrolase |
| 10,60 | aatggtctggaccaga | -112 | SCO6012c | secreted chitinase |
| 10,35 | aatcgtcaagacctgt | -117 | SCO3490 | transposase |
| 9,95 | agaggtcaagatcact | -102 | SCO4506 | unknown |
| 9,70 | ccttgtctagaccaat | -160 | SCO7224c | integral membrane protein |
| 9,45 | actggcggagacctct | -129 | SCO1906c | secreted protein |
| 9,23 | tgttgtctagtccaat | -313 | SCO3679 | unknown |
| 9,09 | tctggtctagtcctgg | -119 | SCO5230c | integral membrane protein |
| 9,01 | taaggtctagacctgc | -113 | SCO6345 | unknown |
